# P-1846. Education and Point-of-Care Testing to Improve Hepatitis A Vaccination Rates Among Future Food-Related Workers

**DOI:** 10.1093/ofid/ofaf695.2015

**Published:** 2026-01-11

**Authors:** Jun Hwi Cho, Jin Kim, Ran Lee, So Hyun Bae, Jang Gwon Yoon, Ji In Seo, Hye Rin Na, A Ram Park, Kyung-Hwa Park, So Yeon Ryu, Seong Eun Kim

**Affiliations:** Gwangju Center for Infectious Diseases Control and Prevention, Buk-gu, Kwangju-jikhalsi, Republic of Korea; Gwangju Center for Infectious Diseases Control and Prevention, Buk-gu, Kwangju-jikhalsi, Republic of Korea; Gwangju Center for Infectious Diseases Control and Prevention, Buk-gu, Kwangju-jikhalsi, Republic of Korea; Gwangju Center for Infectious Diseases Control and Prevention, Buk-gu, Kwangju-jikhalsi, Republic of Korea; Gwangju Center for Infectious Diseases Control and Prevention(GCIDC), Buk-gu, Kwangju-jikhalsi, Republic of Korea; Gwangju Center for Infectious Diseases Control and Prevention(GCIDC), Buk-gu, Kwangju-jikhalsi, Republic of Korea; Gwangju Center for Infectious Diseases Control and Prevention(GCIDC), Buk-gu, Kwangju-jikhalsi, Republic of Korea; Gwangju Center for Infectious Diseases Control and Prevention, Buk-gu, Kwangju-jikhalsi, Republic of Korea; Chonnam National University Medical School, GwangJu, Kwangju-jikhalsi, Republic of Korea; Chosun University Medical School, Dong-gu, Kwangju-jikhalsi, Republic of Korea; Chonnam National University Medical School, GwangJu, Kwangju-jikhalsi, Republic of Korea

## Abstract

**Background:**

Hepatitis A virus (HAV) infection remains a public health concern in South Korea, with periodic outbreaks mainly affecting young adults. However, adults in their 20s to 40s continue to demonstrate low anti-HAV seropositivity rates, with only 19.5% in their 20s and 32.4% in their 30s (Lee et al., 2021). Strategies to identify susceptible adults and enhance vaccination uptake are needed.

**Figure:**
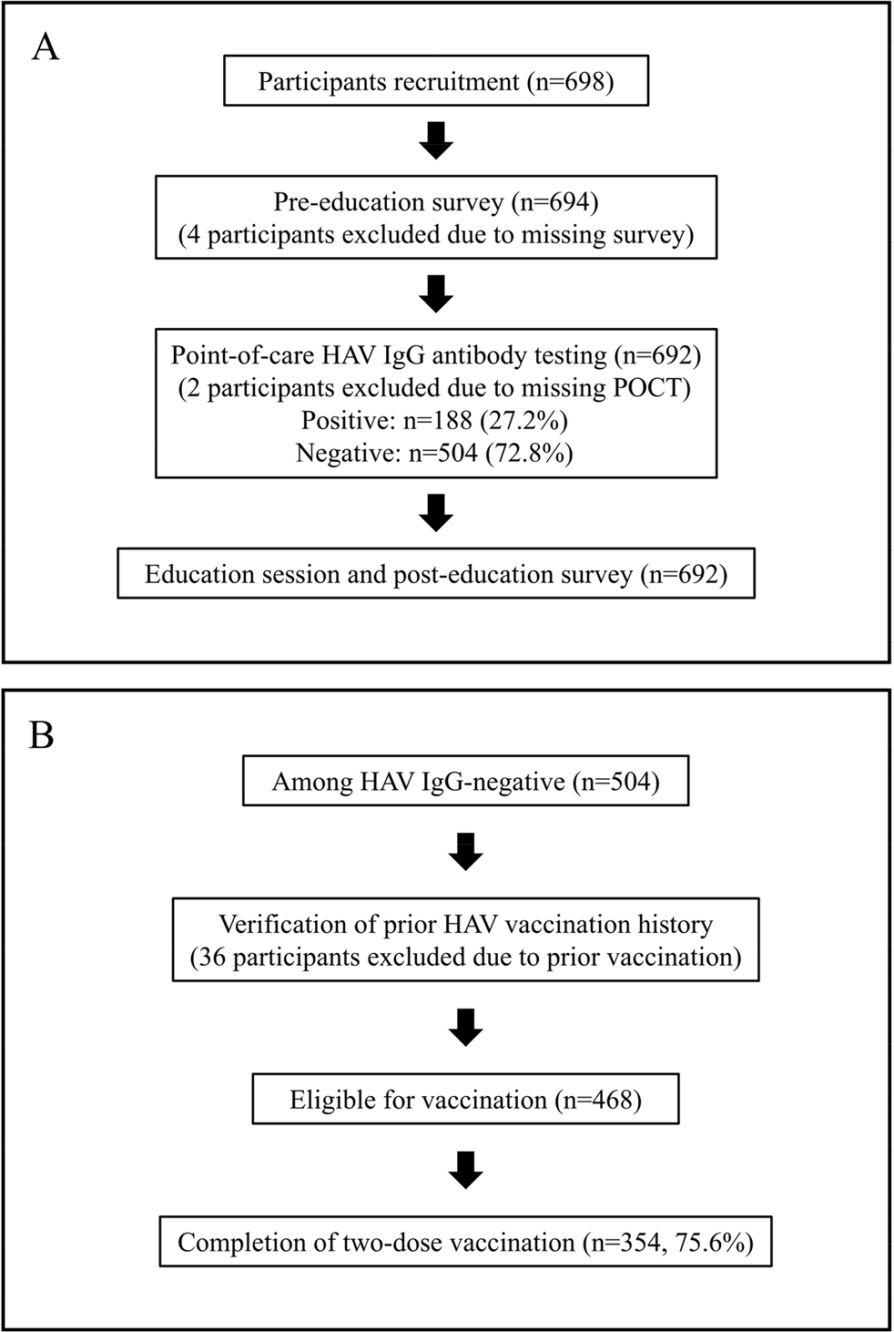
Flowchart of participant enrollment, testing, and vaccination process. Flow diagram of participant inclusion, antibody testing, and vaccination completion. Panel A shows the testing and educational flow for all enrolled students. Panel B describes the vaccination process among those with negative anti-HAV IgG, including exclusion of those previously vaccinated and completion rates for two-dose HAV vaccination.

**Methods:**

We conducted a prospective observational study involving 692 college students from food-related departments in Gwangju, South Korea, between 2023 and 2024. Participants completed pre- and post-education surveys assessing hepatitis A awareness, knowledge, and preventive behaviors. Point-of-care HAV IgG antibody testing (POCT) was performed using the *BIOCREDIT HAV IgG Antibody Diagnostic Kit* (Rapigen Inc., Suwon, South Korea). Factors associated with HAV antibody positivity and vaccination completion were analyzed.

**Results:**

The HAV IgG seropositivity rate was 27.2%. In multivariate analysis, participants who had completed military service as cooks were significantly more likely to be seropositive compared to those without military service (adjusted OR (aOR) 27.1, 95% CI: 2.94 - 250.02, p=0.004). Among 468 vaccination-eligible individuals identified by POCT and vaccination history, 354 (75.6%) completed HAV vaccination. In this group, those who expressed vaccination intent (aOR 3.5, 95% CI: 2.15 - 5.73, p< 0.001) and showed improvement in vaccine understanding post-education (aOR 1.1, 95% CI: 1.01 - 1.26, p=0.032) were significant predictors of vaccination uptake.

**Conclusion:**

Although previous studies have reported that education can improve vaccination uptake, its impact has often been limited. This study suggests that combining point-of-care antibody testing with education not only facilitates the identification of vaccination-eligible individuals but also may contribute to a higher vaccination completion rate.

**Disclosures:**

All Authors: No reported disclosures

